# Circadian Characteristics in Patients under Treatment for Substance Use Disorders and Severe Mental Illness (Schizophrenia, Major Depression and Bipolar Disorder)

**DOI:** 10.3390/jcm10194388

**Published:** 2021-09-25

**Authors:** Ana Belén Serrano-Serrano, Julia E. Marquez-Arrico, José Francisco Navarro, Antonio Martinez-Nicolas, Ana Adan

**Affiliations:** 1 Department of Clinical Psychology and Psychobiology, School of Psychology, University of Barcelona, Passeig de la Vall d’Hebrón 171, 08035 Barcelona, Spain; anik_83@hotmail.com (A.B.S.-S.); jmarquez@ub.edu (J.E.M.-A.); 2 Department of Psychobiology, Campus de Teatinos s/n, School of Psychology, University of Málaga, 29071 Málaga, Spain; navahuma@uma.es; 3 Chronobiology Lab, Department of Physiology, Mare Nostrum Campus, IMIB-Arrixaca, IUIE, College of Biology, University of Murcia, 30100 Murcia, Spain; antilas@um.es; 4 Human Physiology Area, Faculty of Sport Sciences, University of Murcia, Santiago de la Ribera-San Javier, 30720 Murcia, Spain; 5 Ciber Fragilidad y Envejecimiento Saludable (CIBERFES), 28029 Madrid, Spain; 6 Institute of Neurosciences, University of Barcelona, 08035 Barcelona, Spain

**Keywords:** circadian rhythm, dual disorders, chronobiology, substance use disorders, schizophrenia, major depressive disorder, bipolar disorder, distal skin temperature

## Abstract

Dual disorders (substance use and mental illness comorbidity) are a condition that has been strongly associated with severe symptomatology and clinical complications. The study of circadian characteristics in patients with Severe Mental Illness or Substance Use Disorder (SUD) has shown that such variables are related with mood symptoms and worse recovery. In absence of studies about circadian characteristics in patients with dual disorders we examined a sample of 114 male participants with SUD and comorbid Schizophrenia (SZ+; *n* = 38), Bipolar Disorder (BD+; *n* = 36) and Major Depressive Disorder (MDD+; *n* = 40). The possible differences in the sample of patients according to their psychiatric diagnosis, circadian functioning with recordings of distal skin temperature during 48 h (Thermochron iButton^®^), circadian typology and sleep-wake schedules were explored. MDD+ patients were more morning-type, while SZ+ and BD+ had an intermediate-type; the morning-type was more frequent among participants under inpatient SUD treatment. SZ+ patients had the highest amount of sleeping hours, lowest arousal and highest drowsiness followed by BD+ and MDD+, respectively. These observed differences suggest that treatment for patients with dual disorders could include chronobiological strategies to help them synchronize patterns with the day-light cycle, since morning-type is associated with better outcomes and recovery.

## 1. Introduction

As defined by the Word Health Organization in its lexicon of alcohol and drug terms, Dual Disorders are defined as the comorbidity of at least one Substance Use Disorder (SUD) and one Severe Mental Illness (SMI) in the same person [1], being the most frequent psychotic, bipolar and depressive spectrum disorders [2,3]. Given the heterogeneous nature, high prevalence and clinical and functional implications of dual disorders, in recent years interest in its study has increased with the aim of improving both the detection and the therapeutic approach [2,3,4].

Different studies have shown that addictive behavior has negative effects on circadian rhythmic expression [5,6] which can persist weeks or months after starting substance withdrawal [7,8] and they do not always respond to treatment with medication [9,10] Rhythmic alterations commonly observed in patients with SUD are amplitude reduction and phase delay, which in severe cases can lead to chronodisruption or disappearance of rhythmicity [8]. This affectation is related both to the type of substance used and to the person’s metabolism and tolerance (i.e., sensitivity to reward) [11].

The relationship between circadian rhythms and SUD is bidirectional, with evening-type as a precipitating factor for drug use and with drug use generating chronodisruption [8,9]. Moreover, sleep disturbances have been associated with a higher risk of drug use and relapses [12,13], while the magnitude of the phase delay with the degree of dependence [14]. Thus, the exploration of the affectation and recovery of circadian rhythmicity seems to be of special relevance in patients under treatment for SUD [7,15]. In relation to the circadian typology, there seem to be rhythmic differences depending on the chronotype. The evening typology has been identified as a probable risk factor for the development of SUD, while the morning typology has been identified as a protective factor [16,17]. The reinforcing effect of the substances, mediated by clock genes, is greater in evening typology, especially during adolescence and early youth [16,18].

Studies in patients with SUD who have completed a detoxification phase indicated they have a higher prevalence of the morning and intermediate typologies, compared with those who have not initiated treatment, which seems to be a positive characteristic linked to better adherence to treatment [7,15]. Likewise, these patients exhibited a more robust circadian pattern of distal skin temperature (DST) than healthy controls, which is associated with longer abstinence periods and related to some of the treatment strategies used in therapeutic communities (e.g., strong daytime activity and morning habits). Furthermore, poorer sleep quality in patients with SUD is linked to their age and to a greater fragility of the circadian rhythm and chronodisruption [15].

There is a great lack of knowledge about the circadian characteristics in patients with SUD and comorbid SMI. Thus, to date no study has been conducted in dual patients with the diagnoses of schizophrenia (SZ+), bipolar disorder (BD+) and major depressive disorder (MDD+). In this sense, the available evidence on circadian rhythmicity in psychiatric conditions without SUD is limited and heterogeneous, indicating disruptions in prodromal phases in patients without medication [19,20] and in those with remission symptoms or in a sustained withdrawal phase [15,21,22,23]. All of this evidence suggests that circadian alterations could not only be symptoms but a significant clinical characteristic that affects the appearance and development of SMI [24,25].

Patients with schizophrenia show phase delays, free-running rhythms of 48 h or less than 24 h in the sleep-wake cycle or in melatonin secretion, flat amplitude and fragmentation of the activity-rest pattern [26,27,28,29]. All these alterations could be related to poor endogenous control and/or inadequate exposure to external synchronizers [28]. On the other hand, disruptions in the sleep-wake rhythm in schizophrenia have been associated with both clinical and functional prognosis. Deficits in sleep quality have been related with the predominance of positive symptoms [28,30,31] and a lower amplitude and inter-daily stability in the activity-rest rhythm, with poorer neurocognitive performance [27]. Some authors have observed an evening typology predominance in these patients compared to healthy controls [32,33], while in other work no differences have been appreciated [34]. None of these studies found a relationship between circadian typology and the age of the patient, as observed in healthy control subjects. Overall, no previous work has evaluated the circadian rhythm of DST in patients with schizophrenia.

Regarding patients with bipolar disorder, the findings indicate a reduced amplitude activity-rest rhythm, with phase delays (for example, melatonin secretion), greater rhythm fragmentation and more prevalent evening-type [35,36]. Furthermore, although depressive and maniac mood episodes change the rest and activity patterns, circadian alterations persist in euthymic phases [22], which seems to be relevant for the differential diagnosis and for non-psychiatric groups at risk of bipolar disorder [37,38].

Stabilized bipolar patients do not differ in nocturnal activation but they do show less intradaily variability compared to control participants, which may be due to depressive symptoms dependent on variable mood [23,39]. Two systematic reviews that examined activation and energy patterns found that both the euthymic and depressed bipolar groups differ from the controls by a lower mean activity mediated by mood, also concluding that it may be a consequence of bipolar disorder itself [21,40]. It is remarkable that the results that point out a delayed circadian phase in these patients are associated with a younger age, a shorter duration of bipolar disorder and more frequent depressive episodes [41], while those patients who show an advanced phase presented manic episodes and more suicide attempts [42].

Finally, in relation to circadian characteristics in MDD+ patients, only one published study [15] compared these dual patients with SUD ones and explored the possible influence of outpatient vs. residential treatment in therapeutic community. Such work described that the SUD group in therapeutic community presented a better adjustment to the light-dark cycle and a better DST pattern (greater amplitude, relative amplitude and percentage of rhythm and lower minimum temperature average) compared with MDD+ and with patients under outpatient treatment. Furthermore, the therapeutic community patients had the highest prevalence of morning-type regardless of their psychiatric diagnosis. These observations contrast with studies that have described an association of the evening typology with SUD [6,14] and with depression [43,44], although in none of these studies the participants were under residential treatment.

Even though the circadian rhythmic alteration or even its chronodisruption are not precipitating factors for mental disorders, they are related to a greater clinical symptomatology, more difficulties for remission, worse clinical prognosis, lesser healthy habits and worse quality of life [6,45]. All of this can be applicable to both SUD and the three comorbid diagnoses (SZ+, BD+ and MDD+) that have focused our attention on this study.

Therefore, the main goal of this work is to explore the possible differences in circadian rhythmicity in a sample of under treatment patients with SUD taking into account their comorbid SMI. Additionally, we aim to elucidate the possible relationship among circadian rhythmicity with epidemiological and clinical characteristics. This research could provide data of interest and applicability at the therapeutic level, especially when it comes to improve treatment adherence and recovery of dual patients with different SMI.

## 2. Materials and Methods

### 2.1. Study Design and Participants

The present study has a cross-sectional multicenter design, with a sample of 114 male patients with a diagnosis of dual disorder undergoing treatment for SUD (outpatient or residential) in different public and private specialized services located in the province of Barcelona. All patients with SUD were divided into three groups according to the comorbid psychiatric diagnosis: SZ+ (*n* = 38), BD+ (*n* = 36) and MDD+ (*n* = 40).

The inclusion criteria for the study were: (a) men between 20 and 50 years of age; (b) diagnosis of SUD (dependence) in initial remission phase according to Diagnostic and Statistical Manual of Mental Disorders (DSM-5) criteria [46] (c) comorbid psychiatric diagnosis of Schizophrenia, Bipolar Disorder or Major Depressive Disorder, not induced by substances or due to medical condition and (d) a minimum abstinence period of three months up to one year. The consideration of including only men patients in our sample is based on the higher prevalence of SUD in this gender and due to significant greater proportion of men in treatment facilities [2]. Moreover, only males were included to avoid possible biases in the circadian characteristics generated by the differential consumption patterns observed in men vs. women [2]. On the other hand, the exclusion criteria were: (a) patients with unstable or uncontrolled psychiatric symptoms or (b) inability (intellectual, cognitive, developmental or physical) to complete the assessment. Disorders related to caffeine and nicotine consumption were not considered as SUD, although data related to the consumption of both substances were recorded.

For the comparison of temperature data, a group of 40 male healthy control (HC) volunteers (mean age 36.50 yrs.; SD = 8.83; age range 21–51 yrs. old) recruited and assessed at the University of Murcia was also included. Regarding these participants 62.5% were married/with a stable partner, 25% were single and 12.5% were separated/divorced; the majority of them were active (working 88%), and very few were unemployed (10%) or with a disability pension (2%). None of the participants in the HC group had a medical or psychiatric diagnosis, nor any past or present SUD, and they were not under any kind of pharmacological treatment.

This study was approved by the Ethics Committee of the University of Barcelona (registration number: IRB00003099) and complied with the ethical principles of the Declaration of Helsinki. Participation in the study was voluntary and the patients did not receive any compensation except for individualized verbal feedback of their results.

### 2.2. Sociodemographic and Clinical Assessment Instruments

Sociodemographic and clinical data were collected through the Structured Clinical Interview for Axis I Disorders of the DSM-IV (SCID-I) [47], together with a structured interview specifically designed for our study. All collected data were corroborated by the psychologist/psychiatrist in charge of patient’s treatment, as well as checked in the clinical records of each treatment center.

The Positive and Negative Syndrome Scale (PANSS) [48] in its Spanish version was used [49] for the assessment of psychiatric symptoms in the SZ+ group. The PANSS scale yields scores in four areas related to different symptomatology: positive syndrome, negative syndrome, composite scale and general psychopathology. In the BD+ group, we used the Young Mania Rating Scale (YMRS) [50] to measure the severity of maniac symptoms as well as the Hamilton Depression Rating Scale (HDRS) [51]. The YMRS in its Spanish version [52] gives a total score from 0 to 60 understood as it follows: 0–6 euthymic, 7–20 mixed episode and >20 possible maniac episode. On the other hand, the HDRS was used to assess depressive symptoms for the BD+ and MDD+ groups, with its 17-item Spanish version [53] and cut-off points being: 0–7, no current depression; 8–13, low; 14–18, mild; 19–22, severe; and >23, very severe depressive symptoms.

### 2.3. Circadian Assessment Instruments

To evaluate the circadian typology, the Spanish version of the Composite Morning Scale (CSM) [54] was used, consisting of 13 items and a total score from 0 to 55. Its interpretation considers the following cut-off points: 13–25 as an evening typology, 26–36 as an intermediate typology and 37–55 as a morning typology. The sleep-wake schedules were recorded using the structured interview designed for our study.

DST was recorded using the Thermochron iButton^®^ DS1921H (Maxim Integrated Products, San Jose, CA, USA), previously programmed to take measurements every 2 min for 48 consecutive hours with an accuracy of ±0.125 °C. The sensor, which is attached to a strap similar to that of a wristwatch, was placed on the wrist of the non-dominant hand over the temporal artery [55].

### 2.4. Statistical Analysis

For the sociodemographic, clinical and SUD data, descriptive statistics were calculated for the three groups of patients (mean, standard deviation, frequencies and percentages) and subsequent contrasts were performed with ANOVA and Chi-square, depending on the data were parametric or non-parametric.

For the analysis of the DST data, the CircadianwareTM software version 7.1.1 [56] was used. The parametric analyses of cosinor (maximum and minimum temperature, mesor, amplitude, acrophase and percentage of variance explained by the cosine wave), and the analysis of the Rayleigh vector and the Fourier analysis with the first 12 harmonics were made to characterize the circadian rhythm of the DST. The circadianity index was calculated as detailed in previous publications [57]. Non-parametric analyses were performed [55,58] to obtain the values of interdaily stability (IS), intradaily variability (IV), relative amplitude (RA), maximum mean temperature in 5 consecutive hours (M5), temperature minimum average in 2 and 10 consecutive hours (L2 and L10).

DST values, both parametric and non-parametric, and sleep schedules (after transformation to the centesimal system) were evaluated using MANCOVA, while IS and CSM scores were analyzed with ANCOVA. In all cases, age was considered as a covariate, the analyses were performed with the diagnostic group as a factor (SZ+, BD+ and MDD+) and they were repeated considering treatment modality (outpatient/residential). Furthermore, in the parametric analyses for the DST the HC group was also incorporated together with the three clinical groups. Correlational analyses were also performed among DST and clinical variables. Subsequently, a linear regression analysis was carried out with significant correlations at the level of *p* = 0.01. The effect size was calculated as an estimate of the risk of committing type I error with the partial square index of Eta (*ηp*^2^), assuming values of 0.01 as low, 0.06 as moderate and 0.14 as high [59]. Bonferroni test was applied in all the post-hoc contrasts. The data of the present study have been analyzed using the Statistical Package for the Social Sciences program (IBM SPSS Statistics 25.0, Armonk, New York, United States). Two-sided statistical significance was established with a predefined type I error of 5% (*p* < 0.05).

## 3. Results

### 3.1. Sociodemographic and Clinical Characteristics

The groups showed significant differences (see Table 1) in the sociodemographic variables studied such as age (*p* = 0.009), marital status (*p* = 0.011), number of children (*p* = 0.002), family situation (*p* = 0.001), employment status (*p* = 0.001) and years of study (*p* = 0.026). The mean age of the total sample of patients was 37.72 yrs. old (SD = 7.68), observing a higher mean age in SZ+ with respect to MDD+ (*p* = 0.005). The comparison among the three groups and HC subjects did not show differences for age (F_(3,153.)_ = 3.176; *p* = 0.167) while they did for marital status (χ^2^_(3)_ = 39.771; *p* < 0.001), economic situation (χ^2^_(3)_ = 125.086; *p* < 0.001) and years of schooling (F_(3,153)_ = 8.903; *p* < 0.001).

The analysis of the clinical variables (see Table 2) confirmed that a high percentage of patients with MDD+ were under a residential treatment in therapeutic community, in contrast with the SZ+ (χ^2^_(1)_ = 19.90; *p* = 0.001) and BD+ groups (χ^2^_(1)_ = 8.85; *p* = 0.003) who were receiving an outpatient follow-up. The SMI age of onset was later in the MDD+ group compared with SZ+ (*p* = 0.001) and to BD+ (*p* = 0.019). In contrast, no differences were found among the groups for medical disease comorbidities, family history and years of duration of the SMI, and global functioning (not showed in table). Regarding pharmacological treatment, the SZ+ group took a greater amount of psychotropic daily drugs than the MDD+ group (*p* = 0.026), while the BD+ group was in an intermediate position. In all groups the percentage of smoking patients was >80%, with no differences in the active smoking years (>17 years; not showed in table). The mean score of the Fagerström questionnaire for nicotine dependence was higher in the SZ+ group compared to MDD+ (*p* = 0.008). Instead, the caffeine daily intake did not exhibit differences among the groups (not showed in table). Scores on the PANSS, Hamilton and YMRS clinical scales indicated that all groups were clinically stable, although the MDD+ group (11.23 ± 5.14) had a higher HDRS score than the BD+ group (6.86 ± 5.17) (F_(1,74)_ = 5.44; *p* = 0.024).

The results in the clinical variables related to SUD did not indicate any differences among groups for the SUD age of onset, but they did in its duration, being higher in MDD+ patients compared to SZ+ (*p* = 0.05). The SZ+ group consumed more amounts of substances compared to BD+ (*p* = 0.001) and MDD+ (*p* = 0.037), although we obtained a majority pattern of polydrug use in the entire sample (>80% in each group; not showed in table), regardless of the SMI diagnosis. The most commonly used substances in all groups were cocaine, alcohol and cannabis. The DAST-20 revealed a higher proportion of MDD+ patients with high and severe dependence compared to BD+ (χ^2^_(3)_ = 7.83; *p* = 0.035). The groups did not contribute differences in the abstinence period, or in the number of relapses prior to the start of treatment. The presence of family, work and legal related problems did not exhibit differences either (not showed in table).

### 3.2. Circadian Typology and Sleep-Wake Data

The ANCOVA analysis (see Table 3) showed a mean score for the CSM questionnaire in the morningness range for the MDD+ group (*p* = 0.026; ηp^2^ = 0.064), in contrast with for patients with SZ+ (*p* = 0.008) and BD+ (*p* = 0.002), who were placed in the intermediate range. The percentage of patients in the morning typology was also higher in the MDD+ group compared to the SZ+ (χ^2^_(2)_ = 9.60; *p* = 0.008) and BD+ (χ^2^_(2)_ = 7.81; *p* = 0.02) groups. In both SZ+ and BD+ the predominating typology was the intermediate one. Regarding sleep-wake schedules, a greater total sleep duration was observed for SZ+ patients compared to MDD+ (*p* = 0.001); without differences from the BD+ group, which showed an intermediate position. Furthermore, the MDD+ group got up earlier than SZ+ (*p* = 0.009) and BD+ (*p* = 0.015).

On the other hand, the mean score on the CSM scale according to the type of treatment (outpatient vs. residential) provided a similar score for both modalities. However, the percentage of morning typology patients was higher in the residential treatment modality than in the outpatient one (*p* = 0.004). Being under an outpatient treatment program was associated to the intermediate circadian typology. The proportion of people with evening chronotype was also the minority for both treatment modalities. The influence of the type of treatment on sleep-wake schedules showed that outpatients slept for more hours (*p* = 0.001), went to bed and got up later (*p* = 0.006 and *p* = 0.001, respectively). Furthermore, the percentage of patients who took naps and its duration was also higher in the outpatient treatment group (*p* = 0.001).

### 3.3. Distal Skin Temperature

**Figure 1:**
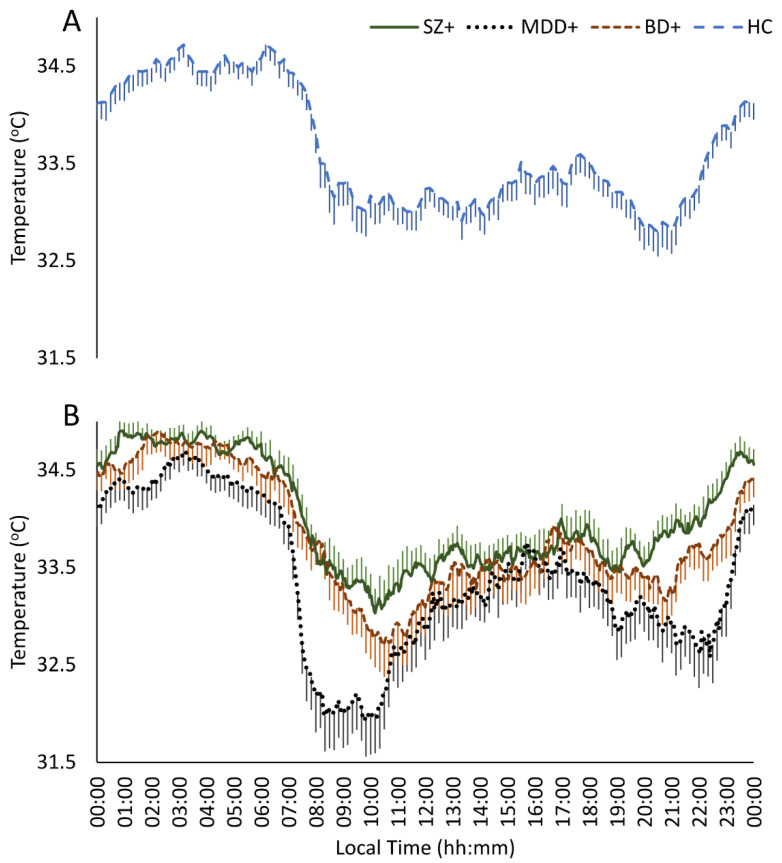
Distal skin temperature mean waveforms for healthy controls (**A**) and dual disorders (**B**). Waveforms data are expressed as mean ± SEM in function of local time (hours:minutes). HC: Healthy Controls (dashed blue line, *n* = 40); SZ+: Substance use disorder with comorbid schizophrenia (dashed red line, *n* = 38), MDD+: Substance use disorder with comorbid major depressive disorder (dotted black line, *n* = 40), BD+: Substance use disorder with comorbid bipolar disorder (continuous green line, *n* = 36).

Table 4 shows the results of the DST analyses for the three groups of patients and the data for the HC group. The MANCOVA carried out, both for the parametric and non-parametric indexes, showed significant differences depending on the comorbid SMI diagnoses (see Figure 1). The SZ+ group presented the highest minimum and mesor compared to the MDD+ group (*p* = 0.021), without differences from the BD+ group, which was in an intermediate position. We also found a higher M5 value for BD+ patients compared with MDD+ (*p* = 0.025).

The comparison of the DST parameters between the dual groups and the HC group showed a higher minimum value (*p* = 0.002) and mesor (*p* = 0.024) in the SZ+ group, while the Rayleigh vector (*p* = 0.036) and the accumulated potency of the first 12 harmonics (*p* = 0.039), were higher in the MDD+ group. In addition, the CI was found below the value of the HC group and the range of normality for SZ+ (*p* = 0.004) and MDD+ (*p* = 0.001). In the case of IV, the degree of fragmentation was practically null in the three dual groups and significantly different from the HC group (*p* = 0.001, in all cases). Finally, BD+ patients showed a higher M5 value than the HC group (*p* = 0.011), although the value of this parameter in the three groups indicated an adequate night’s rest (see Figure 1).

**Figure 2:**
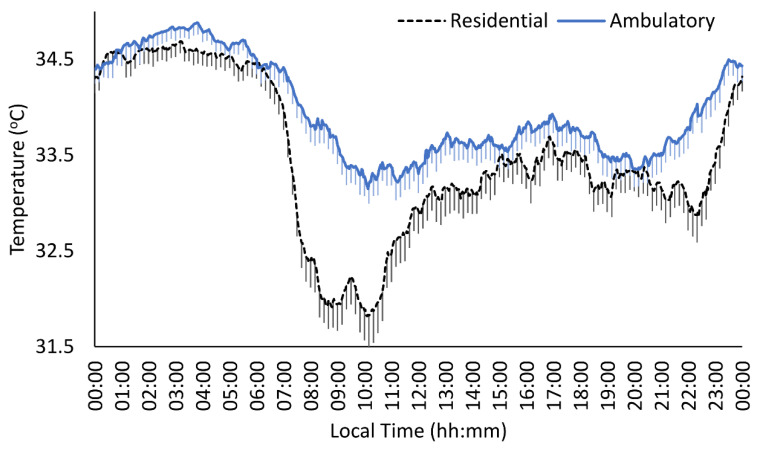
Distal skin temperature mean waveforms for residential treatment (dashed black line, *n* = 50) and ambulatory treatment (continuous blue line, *n* = 60). Waveforms data are expressed as mean ± SEM in function of local time (hours:minutes).

In the additional analyses carried out considering treatment modality as a fixed factor (see Table 5 and Figure 2), the MANCOVA analyses indicated that the dual outpatients presented the highest minimum, mesor and L10 values, and a later acrophase (*p* = 0.007) together with a delay in the central hour of the waking period with respect to those who received residential treatment. The last MANCOVA adding the diagnostic group factor, to determine whether the differences between the type of treatment differed according to the comorbid SMI, did not provide differences in any of the evaluated parameters.

Finally, the results of the correlational analysis (*p* ≤ 0.01) between the DST and the clinical variables showed significant relationships with nicotine dependence. In this sense, the SZ+ group exhibited a negative relationship for the values of mesor and L10, and the Fagerström score (r = −0.469; *p* = 0.003 and r = −0.438; *p* = 0.007, respectively), that is, the higher the nicotine dependence score the lower values of both were observed. Likewise, in the MDD+ group, a negative correlation was obtained between M5 and the daily amount of cigarettes consumed (r = −0.475; *p* = 0.004); thus, in these patients, the higher the consumption of nicotine, the lower the temperature values in the five hours of maximum value.

## 4. Discussion

This study aims to analyze differences in circadian rhythmicity in patients under treatment with SUD attending to comorbid SMI, as well as its possible relationship with epidemiological and clinical characteristics.

Regarding sociodemographic and clinical results, the three groups of dual patients showed characteristics in line with previous studies [5,60,61,62]. Our results indicated an important presence of factors related to a worse clinical symptomatology and prognosis, especially for patients in the SZ+ group, and are consistent with the available literature [63,64,65]. Moreover, for patients with SZ+ and BD+, the outpatient treatment modality was predominant, while for patients with MDD+ the residential and therapeutic community treatment was more frequent. On the other hand, the mean age of SMI onset was earlier for the SZ+ group, this observation has been associated with a worse clinical, cognitive and functional prognosis [60,66,67]. As in previous studies, our sample presented similarities in psychiatric family history, medical disease comorbidity and previous suicide attempts [68,69,70]. Regarding nicotine consumption, although the three groups showed a high percentage of smokers and a moderate level of dependence, the SZ+ group exhibited the highest consumption and the MDD+ the lowest one.

Moreover, the longer duration of the SUD in patients with MDD+ may be related to the older age of the group, greater latency from the onset of depression until they seek professional help or may be unsuccessful attempts at previous treatments. According to available publications, in the three groups the most commonly used substances were cocaine, alcohol and cannabis [69,71,72], with an important common pattern of polydrug use in all the groups [4,60,69,72]. While the main substance of abuse may have a specific role in clinical and circadian rhythmic variables, polydrug use as a common pattern makes difficult to address such specific analysis. On the other hand, the severity of addiction was higher in the SZ+ group (followed by MDD+) in association with the consumption of a greater number of substances [73]. All these indicators would confirm the need for continuous, comprehensive treatments that affect relapse prevention [70].

Regarding the circadian typology, and in line with a previous study [15], in the MDD+ group the morning typology was the predominant one while in the SZ+ and BD+ groups the intermediate typology was the most frequent. The highest percentage of people with morning typology was observed in patients receiving residential treatment vs. outpatient modality. This observation is consistent with previous studies [14,15,43] and points out that there could be a possible regulating effect of the circadian rhythm generated by the habits and routines imposed by a residential treatment. The restoration of an adequate circadian rhythm is an element that contributes to the clinical improvement of patients with major depression [44], and according to our results, this also could be extended to those with MDD+, SZ+ and BD+.

On the other hand, in agreement with previous data in patients with a single SMI diagnosis, such as schizophrenia and bipolar disorder [74], the total duration of daily sleep in SZ+ patients were higher than those with MDD+, placing patients with BD+ in an intermediate position. This could be explained by the delay in getting up observed in patients with SZ+, which may be related to the sedative effect of most typical/atypical antipsychotic drugs and anticholinergics they were taking [66]. Likewise, those who received outpatient treatment slept more hours a day, went to bed and got up later, and took more and longer naps. This suggests that sleep-wake rhythm time imbalances are not simply a consequence of clinical symptoms and could be influenced by treatment modality. The morningness tendency can be considered a marker of adherence to treatment and as a protective factor for relapses in both SUD and depressed patients [14,15,75]. Treatments that enhance synchronization with the environmental signals of the light-dark cycle, work on the regularity of schedules and include practice of physical exercise influence circadian recovery [19,25]. Our findings emphasize need to incorporate chronobiological adjustment strategies in dual patients under outpatient treatment modality, especially in the cases of those with SZ+ or BD+.

Regarding the circadian pattern of DST, it indicates less activation and/or greater daytime sleepiness in SZ+ patients and, to a lesser extent, also in BD+ patients. Even though there are no published data on DST in SZ+ patients, our findings are consistent with studies that have evaluated circadian functioning in patients with a diagnosis of schizophrenia only, where a lower amplitude and greater fragmentation of the activity-rest pattern were also observed [26,28,29]. In addition, we found a higher M5 value in BD+ patients compared to MDD+, that, together with a greater stability of the rhythm, points out a better night’s rest [76] in BD+ patients. However, in the three groups of our sample this data was found within normality values according to population norms. The inclusion of the HC group widened the differences found in the minimum and mesor values, Rayleigh, P12, IC, IV and M5. The SZ+ group obtained a significantly higher minimum and mesor value that denotes a lower diurnal activation [76] compared to control subjects and with the MDD+ group, without differences from BD+.

It is worth mentioning the differential relationship observed between tobacco consumption and circadian rhythmicity [14] regarding the comorbid SMI. For SZ+ patients nicotine dependence was associated to the quality of wakefulness, while for patients with MDD+ nicotine dependence was linked to the sleep period (M5). Thus, tobacco consumption and its level of dependence could be considered as a modulating factor of circadian rhythmicity, which is also related to the type of SMI diagnosis. Even though this should be deepened in the future, smoking seems to impair the quality of sleep for MDD+ patients. Furthermore, the poorer quality of wakefulness shown by patients with SZ+ is minimized in those who smoke, which could be explained by the palliative effect of nicotine over the side effects of antipsychotic treatment.

On the other hand, we found more stability of the circadian rhythm (Rayleigh vector and the power of the first 12 upper harmonics) in the MDD+ group than in the HC group. A previous study [15] observed that superior stability occurred in patients in therapeutic community (residential treatment) vs. those who were in an outpatient program. Therefore, if we take into account that the majority of patients in the MDD+ group underwent treatment in therapeutic community, our results could be congruent due to the probable influence of the type of treatment on circadian rhythmicity. A result to emphasize is the lower rhythm stability index (IC) for patients with SZ+ compared to the HC group, which has been related to a more immature circadian system [58]. Furthermore, the three groups of patients showed a lower IV compared to the HC group. Furthermore, less fragmentation was also found for both the SUD and in the MDD+ groups, regardless of treatment modality [15]. Despite the absence of previous data about rhythm fragmentation in SZ+ and BD+ patients, the alteration of the IV in both cases suggests that it could be used as a psychopathological marker associated with SMIs, as it has been observed in schizophrenia [26,28] and bipolar disorder [23,39] conditions, regardless of the presence of a comorbid SUD.

Regarding treatment modality, our results suggest a better quality of both sleep and wakefulness and a more robust circadian pattern of DST in dual patients under residential treatment. The patients under outpatient treatment, however, showed less daytime activation (minimum, higher L10 value and mesor) and a more evening pattern (later acrophase) compared to patients in residential facilities. Overall, these observations reveal a low contrast in their day-to-day life [77] in consistency with previous observations made in MDD+ [15] and in depression without SUD [44]. Therefore, it is emphasized that the treatment of dual patients, regardless of their SMI comorbidity, should promote rhythmic organization, physical activity in the open air and stable feeding times to maximize a good circadian expression and a morning pattern [14,55,78].

This work has some limitations, such as the cross-sectional design with a sample composed only by men, which rules out the establishment of causal relationships and does not allow the generalization of the results to women. Likewise, the wide age range of the sample, although partially controlled with age as a covariate, might have contributed to type II error. The high pattern of polydrug use in the patients does not allow us to assess specific associations between the type of substance and circadian rhythmicity. On the other hand, the higher proportion of patients in residential treatment in the MDD+ group could have influenced some of the circadian rhythm results attributed to their diagnosis. Future studies should evaluate circadian rhythmicity at the beginning as well as during treatment in order to know the differential evolution of patients and to identify possible risk factors and predictors of therapeutic adherence.

## 5. Conclusions

The present work represents a first contribution to the knowledge of the differential circadian characteristics of dual diagnosis patients taking into account their comorbid SMI, as well as the relationship with sociodemographic, clinical variables and treatment modality received for the SUD. The results obtained point out the importance to consider the circadian rhythmic expression in the clinical management of dual patients, with residential treatment modality being a possible indicator of a better restoration of circadian functioning. Our findings may confirm the idea that the DST rhythm could be a biological marker of treatment adherence and a protective factor of the comorbid SMI disorder; even though future longitudinal studies are required to contrast such evidence. Progress in this line can contribute, firstly, to the detection of possible markers of vulnerability and, secondly, to establishing more appropriate therapeutic goals that incorporate chronotherapeutic strategies. All of this seems especially of interest for patients with greater circadian dysfunction such as those with SZ+, BD+ and under outpatient treatments, in whom the emphasis on maintaining changes in behavior and appropriate time habits may improve the response to treatment and prevent relapses.

## Figures and Tables

**Table 1:** Sociodemographic data for the three groups of patients. Means, standard deviation, percentages and statistical contrasts (ANOVA and Chi Square test).

Sociodemographic Data	SZ+ (*n* = 38)	BD+ (*n* = 37)	MDD+ (*n* = 39)	Contrasts
Age (years)	35.13 ± 8.20	37.58 ± 8.19	40.41 ± 5.67	*F*_(2,111)_ = 4.87 **
Marital status				*χ^2^*_(3)_ = 16.55 *
Single	86.8%	55.6%	47.5%	
Married/stable partner	2.6%	8.3%	10.0%	
Separated/divorced	10.5%	33.3%	42.5%	
Widower	0%	2.8%	0%	
Family situation				*χ^2^*_(1)_ = 11.78 **
Without children	86.8%	61.1%	50.5%	
With children	13.2%	38.9%	50.0%	
Living arrangements				*χ*^2^_(3)_ = 21.88 ***
Alone	7.9%	16.7%	5.0%	
Sharing	65.8%	61.1%	45.0%	
Therapeutic community	15.8%	22.2%	50.0%	
Supported accommodation	10.5%	0%	0%	
Economic situation				*χ^2^*_(3)_ = 26.64 ***
Working	13.2%	11.1%	12.5%	
Unemployed	21.1%	22.52%	55.0%	
Under sick leave	7.9%	5.6%	20.0%	
Disability pension	57.9%	61.1%	12.5%	
Years of schooling	10.00 ± 2.36	11.54 ± 3.07	10.64 ± 2.57	*F*_(2,111)_ = 3.77 *

SZ+: Substance use disorder with comorbid schizophrenia; BD+: Substance use disorder with comorbid bipolar disorder; MDD+: Substance use disorder with comorbid major depressive disorder; * *p* < 0.05; ** *p* < 0.01; *** *p* < 0.001.

**Table 2:** Clinical data for the three groups of patients regarding psychiatric diagnosis and substance use disorders. Means, standard deviation, percentages and statistical contrasts (ANOVA and Chi Square test).

Clinical Data	SZ+ (*n* = 38)	BD+ (*n* = 37)	MDD+ (*n* = 39)	Contrasts
Treament modality				*χ^2^*_(1)_ = 20.93 ***
Outpatient	78.9%	62.2%	28.2%	
Residential	21.1%	37.8%	71.8%	
SMI age of onset (years)	23.53 ± 7.50	26.26 ± 9.75	31.74 ± 8.14	*F*_(2,109)_ = 9.37 ***
Suicide attempts	1.08 ± 1.86	1.38 ± 3.14	0.77 ± 0.98	*F*_(2,111)_ = 0.74
Pharmacological treatment (N/day)	3.35 ± 1.54	3.14 ± 1.82	2.32 ± 1.64	*F*_(2,109)_ = 3.98 *
Typical antipsychotic	26.3%	8.1%	2.6%	*χ^2^*_(1)_ = 11.17 **
Atypical antipsychotic	94.7%	64.9%	12.8%	*χ^2^*_(1)_ = 55.55 ***
Mood stabilizers	36.8%	70.3%	15.4%	*χ^2^*_(1)_ = 23.22 ***
Anxiolytics	42.1%	35.1%	43.6%	*χ^2^*_(1)_ = 0.82
Antidepressants	34.2%	43.2%	71.8%	*χ^2^*_(1)_ = 12.44 **
Anticholinergic	26.3%	2.7%	0%	*χ^2^*_(1)_ = 18.36 ***
Alcohol-aversive-agent	26.3%	21.6%	28.2%	*χ^2^*_(1)_ = 0.56
Other psychotropics	13.2%	13.5%	17.9%	*χ^2^*_(1)_ = 0.47
Chlorpromazine equivalent dose (mg)	406.08 ± 34.73	145.95 ± 35.71	32.44 ± 34.27	*F*_(2,108)_ = 30.65 *
Daily cigarettes per day	21.95 ± 11.54	18.84 ± 8.76	13.23 ± 6.87	*F*_(2,111)_ = 8.80 ***
Fagerström total score	6.11 ± 2.60	5.03 ± 2.75	4.28 ± 2.40	*F*_(2,111)_ = 4.82 **
SUD age of onset (years)	17.24±5.16	20.81 ± 10.10	18.59 ± 7.01	*F*_(2,110)_ = 2.04
Duration of the SUD (years)	17.63 ± 1.42	17.02 ± 1.45	21.82 ± 1.10	*F*_(2,110)_ = 3.39 *
Quantity substances used	3.74 ± 1.44	2.54 ± 1.19	2.95 ± 1.41	*F*_(2,111)_ = 7.55 ***
Type of substance ^a^				
Cocaine	94.7%	64.9%	87.2%	*χ^2^*_(1)_ = 12.47 **
Alcohol	76.3%	89.2%	89.7%	*χ*^2^_(1)_ = 3.46
Cannabis	78.9%	48.6%	53.8%	*χ^2^*_(1)_ = 8.31 *
Ectasis	18.4%	10.8%	5.1%	*χ^2^*_(1)_ = 3.25
Hallucinogens	39.5%	16.2%	20.5%	*χ*^2^_(1)_ = 6.10 *
Opioids	28.9%	13.5%	25.6%	*χ*^2^_(1)_ = 2.87
Anxiolytics/hypnotics	28.9%	10.8%	12.8%	χ^2^_(1)_ = 5.17
DAST-20 total score	12.68 ± 3.18	12.74 ± 9.47	13.74 ± 3.94	*F*_(2,111)_ = 0.30
Severity of addiction				*χ*^2^_(3)_ = 17.58 *
Low	2.6%	10.8%	2.6%	
Mild	13.2%	16.2%	12.8%	
High	42.1%	13.5%	35.9%	
Severe	7.9%	10.8%	28.2%	
Months of abstinence	10.88 ± 1.15	8.44 ± 1.18	7.33 ± 1.14	*F*_(2,111)_ = 2.48
Quantity of relapses	1.05 ± 1.57	0.75 ± 1.30	0.87 ± 1.23	*F*_(2,111)_ = 0.44

SZ+: Substance use disorder with comorbid schizophrenia; BD+: Substance use disorder with comorbid bipolar disorder; MDD+: Substance use disorder with comorbid major depressive disorder; SMI: Severe mental illness; N: Number; SUD: Substance use disorder; DAST-20: Drug abuse screening test. ^a^ Percentages will not equal 100 as each patient may have taken more than one substance. * *p* < 0.05; ** *p* < 0.01; *** *p* < 0.001.

**Table 3:** Circadian typology and sleep-wake data for the three groups of patients. Means, percentages and differences according to the type of treatment.

	SZ+ (*n* = 38)	BP+ (*n* = 37)	MDD+ (*n* = 39)	Contrasts	Outpatient (*n* = 63)	Residential (*n* = 51)	Contrasts
CSM total	34.04 ± 1.11	33.56 ± 1.10	37.55 ± 1.10	*F*_(2,111)_ = 3.77 *	34.14 ± 0.86	36.29 ± 0.97	*F*_(2,112)_ = 2.70
Circadian typology				*χ^2^*_(2)_ = 12.66 *			*χ^2^*_(2)_ = 10.96 **
Morning-type	28.9%	32.4%	64.1%		29.7%	58%	
Intermediate-type	57.9%	48.6%	28.02%		57.8%	28%	
Evening-type	13.12%	18.9%	7.7%		12.5%	14%	
Total sleeping (h)	9.44 ± 0.25	8.75 ± 0.24	8.03 ± 0.24	*F*_(2,112)_ = 7.84 ***	9.03 ± 0.18	7.09 ± 0.20	*F*_(2,112)_ = 24.89 ***
Bedtime	23:07 ± 0.20	23:26 ± 0.20	23:05 ± 0.20	*F*_(2,112)_ = 0.93	23:29 ± 0.15	22:51 ± 0.17	*F*_(2,112)_ = 7.72 **
Getting up time	08:01 ± 0.22	07.93 ± 0.22	07:02 ± 0.22	*F*_(2,112)_ = 5.78 **	08:36 ± 0.15	06:73 ± 0.17	*F_(_*_2,112)_ = 50.97 ***
Nap (yes)	34.2%	24.3%	20.5%	*χ^2^*_(1)_ = 1.97	39.1%	10.0%	*χ^2^*_(1)_ = 12.22 ***
Nap total time (min)	24.93 ± 5.55	13.77 ± 5.03	10.03 ± 5.41	*F*_(2,112)_ = 1.90	25.21 ± 3.96.03	04.22 ± 4.48	*F*_(2,112)_ = 12.99 ***

SZ+: Substance use disorder with comorbid schizophrenia; BD+: Substance use disorder with comorbid bipolar disorder; MDD+: Substance use disorder with comorbid major depressive disorder; * *p* < 0.05; ** *p* < 0.01; *** *p* < 0.001.

**Table 4:** Distal skin temperature for the three groups of patients and the healthy controls group. Means, standard error and MANCOVA analyses.

				MANCOVA			MANCOVA	
	SZ+ (*n* = 38)	BP+ (*n* = 37)	MDD+ (*n* = 39)	*F*_(2,111)_	*ηp* ^2^	HC Group (*n* = 40)	*F*_(3,150)_	*ηp* ^2^
Maximum	36.08 ± 0.09	36.12 ± 0.90	35.98 ± 0.09	0.64	0.01	36.17 ± 0.09	0.98	0.02
Minimum	31.65 ± 0.28	31.16 ± 0.28	30.58 ± 0.28	3.78 *	0.07	30.40 ± 0.21	5.15 **	0.10
Mesor	34.01 ± 0.15	33.83 ± 0.15	33.45 ± 0.15	3.23 *	0.06	33.60 ± 0.09	3.23 *	0.06
Amplitude	0.82 ± 0.13	1.00 ± 0.13	1.10 ± 0.13	1.11	0.02	0.88 ± 0.06	1.08	0.02
Acrophase ^a^	01:30 ± 0.74	00:36 ± 0.74	23:59 ± 0.74	0.97	0.02	02:19 ± 0.43	0.42	0.01
Rayleigh	0.88 ± 0.03	0.88 ± 0.03	0.94 ± 0.03	0.88	0.02	0.80 ± 0.02	2.71 *	0.05
P1	0.56 ± 0.19	0.95 ± 0.19	0.65 ± 0.19	1.15	0.02	0.48 ± 0.07	1.53	0.03
P12	1.01 ± 0.31	1.59 ± 0.32	1.88 ± 0.31	1.83	0.03	0.81 ± 0.11	3.18 *	0.06
CI	0.39 ± 0.03	0.43 ± 0.03	0.34 ± 0.03	1.50	0.03	0.55 ± 0.02	7.47 ***	0.14
IS	0.66 ± 0.02	0.72 ± 0.02	0.72 ± 0.02	1.14	0.02	0.44 ± 0.02	26.45	0.00
IV	0.02 ± 0.00	0.02 ± 0.00	0.02 ± 0.00	0.83	0.02	0.19 ± 0.01	107.56 ***	0.70
RA_10	0.25 ± 0.03	0.32 ± 0.03	0.30 ± 0.03	1.30	0.02	0.26 ± 0.01	1.35	0.03
M5	35.01 ± 0.10	35.12 ± 0.10	34.71 ± 0.10	3.78 *	0.07	34.68 ± 0.07	5.07 **	0.10
TM5 ^a^	01:48 ± 0.72	01:12 ± 0.72	02:03 ± 0.72	0.36	0.01	03:20 ± 0.44	1.81	0.04
L10	33.30 ± 0.22	32.96 ± 0.22	32.66 ± 0.22	1.97	0.04	32.93 ± 0.12	1.80	0.04
TL10 ^a^	16:50 ± 1.03	18:35 ± 1.04	15:16 ± 1.03	2.55	0.05	15:98 ± 0.94	2.06	0.04

SZ+: Substance use disorder with comorbid schizophrenia; BD+: Substance use disorder with comorbid bipolar disorder; MDD+: Substance use disorder with comorbid major depressive disorder; HC: healthy control group; *ηp*^2^: Partial square index of eta (effect size); P1: first armonic power; P12: Cumulative power of the twelfth harmonic; CI: Circadianity index; IS: Interdaily stability; IV: Intradaily variability; RA_10: Relative amplitude multiplied by 10; M5: Mean value of the five consecutive hours of maximum temperature values; TM5: M5 time location; L10: Mean value of the 10 consecutive hours of minimum temperature values; TL10: L10 time location. ^a^ Data expressed in hours and minutes (mean and standard error). * *p* < 0.05; ** *p* < 0.01; *** *p* < 0.001.

**Table 5:** Distal skin temperature for the total sample according to treatment modality. Means, standard error and MANCOVA analyses.

	Treatment Modality		MANCOVA	
	Outpatient (*n* = 63)	Residential (*n* = 51)	F_(2,112)_	*ηp* ^2^
Maximum	36.10 ± 0.06	36.01 ± 0.07	0.74	0.01
Minimum	31.56 ± 0.21	30.54 ± 0.24	9.63 **	0.08
Mesor	33.95 ± 0.11	33.52 ± 0.13	5.64 *	0.05
Amplitude	0.88 ± 0.10	1.08 ± 0.11	1.66	0.02
Acrophase ^a^	01:44 ± 0.55	23:18 ± 0.62	7.59 **	0.07
Rayleigh	0.90 ± 0.02	0.91 ± 0.03	0.05	0.01
P1	0.62 ± 0.14	0.84 ± 0.16	1.03	0.01
P12	1.23 ± 0.24	1.83 ± 0.27	2.66	0.02
CI	0.39 ± 0.02	0.39 ± 0.03	0.00	0.01
IS	0.73 ± 0.02	0.72 ± 0.02	0.23	0.01
IV	0.02 ± 0.00	0.02 ± 0.00	0.60	0.01
RA_10	0.26 ± 0.02	0.32 ± 0.02	2.26	0.02
M5	35.04 ± 0.08	34.83 ± 0.09	2.75	0.02
TM5^a^	01:37 ± 0.55	01:47 ± 0.62	0.04	0.01
L10	33.22 ± 0.16	32.65 ± 0.19	4.88 *	0.04
TL10^a^	18:16 ± 0.78	15:07 ± 0.88	7.01 **	0.07

P1: first armonic power; P12: Cumulative power of the twelfth harmonic; CI: Circadianity index; IS: Interdaily stability; IV: Intradaily variability; RA_10: Relative amplitude multiplied by 10; M5: Mean value of the five consecutive hours of maximum temperature values; TM5: M5 time location; L10: Mean value of the 10 consecutive hours of minimum temperature values; TL10: L10 time location; *ηp*^2^: Partial square index of eta (effect size). ^a^ Data expressed in hours and minutes (mean and standard error). * *p* < 0.05; ** *p* < 0.01.

## Data Availability

The data presented in this study are available on request from the corresponding author.
